# The impact of SGLT2i on the outcome of advanced lung cancer in patients with diabetes

**DOI:** 10.3389/fonc.2026.1759702

**Published:** 2026-02-20

**Authors:** Jindong Chen, Hao Wang, Conghui Shang, Zixu Fan, Ziyi Sheng, Tianqing Chu, Min Zhang, Liang Zhao

**Affiliations:** 1 Department of Cardiology, Shanghai Chest Hospital, School of Medicine, Shanghai Jiao Tong University, Shanghai, China; 2 Department of Clinical Research Unit, Shanghai Chest Hospital, School of Medicine, Shanghai Jiao Tong University, Shanghai, China

**Keywords:** advanced lung cancer, cardiotoxicity, diabetes, outcome, SGLT2i

## Abstract

**Objective:**

Sodium-glucose cotransporter-2 inhibitors (SGLT2i), a novel pharmacological agent for diabetes and heart failure, may influence oncologic outcomes. Their role in advanced lung cancer patients with diabetes is unclear. Our study aims to evaluate SGLT2i’s effects in this population.

**Methods:**

We performed a retrospective analysis of advanced lung cancer patients diagnosed with diabetes at our center between July 2020 and July 2024. The case cohort include patients who received SGLT2i, while the control cohort did not. The primary endpoint was overall mortality, and the secondary endpoint was a composite of cardiac events.

**Results:**

The cohort included 188 patients, with 94 cases and 94 controls. Over a median follow-up of 16 months, the use of SGLT2i was significantly associated with improved survival for advanced lung cancer with diabetes. (hazard ratio, (HR) 0.56; 95% CI 0.35–0.87, P = 0.009). The survival rate was lower in major adverse cardiovascular events (MACE) group than non-MACE group, however the differences were not statistically significant (HR 1.69; 95% CI 0.96– 2.97, P = 0.067). During follow-up, the incidence of MACE (24 cases in total, with new-onset atrial fibrillation/flutter being the most common) did not differ significantly between SGLT2i and control groups (11.7% vs. 13.8%; P = 0.662).

**Conclusions:**

In advanced lung cancer patients with diabetes, SGLT2i was associated with a lower all-cause mortality rate. Yet SGLT2i had insignificant impact on the incidence of MACE and the post-MACE survival.

## 1 Introduction

Lung cancer remains a significant global health challenge, characterized by its high incidence and mortality rates (1). Particularly, lung cancer presents with comorbid conditions such as diabetes mellitus, which can exacerbate the disease’s progression and reduce overall survival, especially in advanced lung cancer (2, 3), which highlighting the need for additional therapeutic strategies to improve outcomes for this vulnerable population.

The glycolytic metabolism of tumor cells plays a pivotal role in lung cancer progression, such as influencing tumor growth, promoting tumor aggressiveness, and contributing to resistance against chemoradiotherapy (4, 5). Sodium-glucose co-transporter 2 (SGLT2) is one of the transporters for active glucose uptake into cancer cells, which has been detected in lung and other cancers (6–8). Sodium-glucose co-transporter 2 inhibitors (SGLT2i), initially developed for managing diabetes, have recently garnered attention for their potential anticancer properties account for inhibiting glucose uptake (8, 9). Although recent studies have suggested that SGLT2i are associated with improved survival in diabetic patients with cancer receiving immune checkpoint inhibitors (10), their impact specifically in advanced lung cancer patients—particularly those undergoing a comprehensive range of anticancer therapies including chemotherapy, targeted therapy, and immunotherapy—on both survival and cardiac safety remains inadequately assessed. Based upon this, our study aims to address this distinct knowledge gap by evaluating the efficacy and safety of SGLT2i in patients with advanced lung cancer and comorbid diabetes.

## 2 Methods

### 2.1 Study population

The present study is a single-center retrospective observational study whose study population selected form the institutional database, and conducted in conformity of the Declaration of Helsinki. The study protocol was approved by Shanghai Chest Hospital Ethics Committee, and written consent was waived. Briefly, the medical records of consecutive patients diagnosed with advanced lung cancer and diabetes between July 2020 and July 2024 were reviewed. Inclusion criteria were as follows. 1) The patients were diagnosed Stage III-IV NSCLC according to World Health Organization (WHO) histological classification and the UICC/AJCC TNM Classification; 2) the advanced lung cancer patients with concurrent diabetes; 3) patients with a score of 0–2 assessed by Eastern Cooperative Oncology Group’s performance status (ECOG PS); Exclusion criteria included: 1) age <18 or ≥80 years old; 2) Survival period shorter than 3 months; 3) incomplete medical records.

### 2.2 Study endpoints

The primary endpoint was all-cause mortality. The secondary endpoint was a composite of major adverse cardiovascular events (MACE), including new-onset atrial fibrillation/flutter, acute coronary syndrome, myocarditis, heart failure and third-degree atrioventricular block or other malignant arrhythmias (sustained ventricular tachycardia or ventricular fibrillation).

### 2.3 Statistical analysis

For normally distributed variables, continuous variables are expressed as mean± standard deviation (SD), and compared by independent t-test. For variables with non-normal distributions, continuous variables were expressed as median (interquartile range, IQR) and compared by Mann-Whitney U test. Categorical variables are expressed as percentage and compared by Chi-square test. A 1:1 propensity score matching was performed to balance the differences between SGLT2i group and control group in baseline characteristics, including age, sex, TNM stage and the pathological types of lung cancer. Matching was based on propensity scores yielded by logistic regression model. The cut-off value of the matching tolerance was set at 0.02 to obtain satisfactory matching. The overall survival of the two groups was compared via Kaplan-Meier methods and log-rank test. Cox proportional hazard models were applied to assess the risk of all-cause mortality between SGLT2i group and control group. A two-tailed P value<0.05 was considered statistically significant. All statistical analyses were performed with the SPSS (IBM SPSS 26.0 IBM Corp, Armonk, NY, USA) and R statistical package 3.3.1 (R Foundation for Statistical Computing, Vienna, Austria).

## 3 Result

### 3.1 Patient characteristics

**Figure 1:**
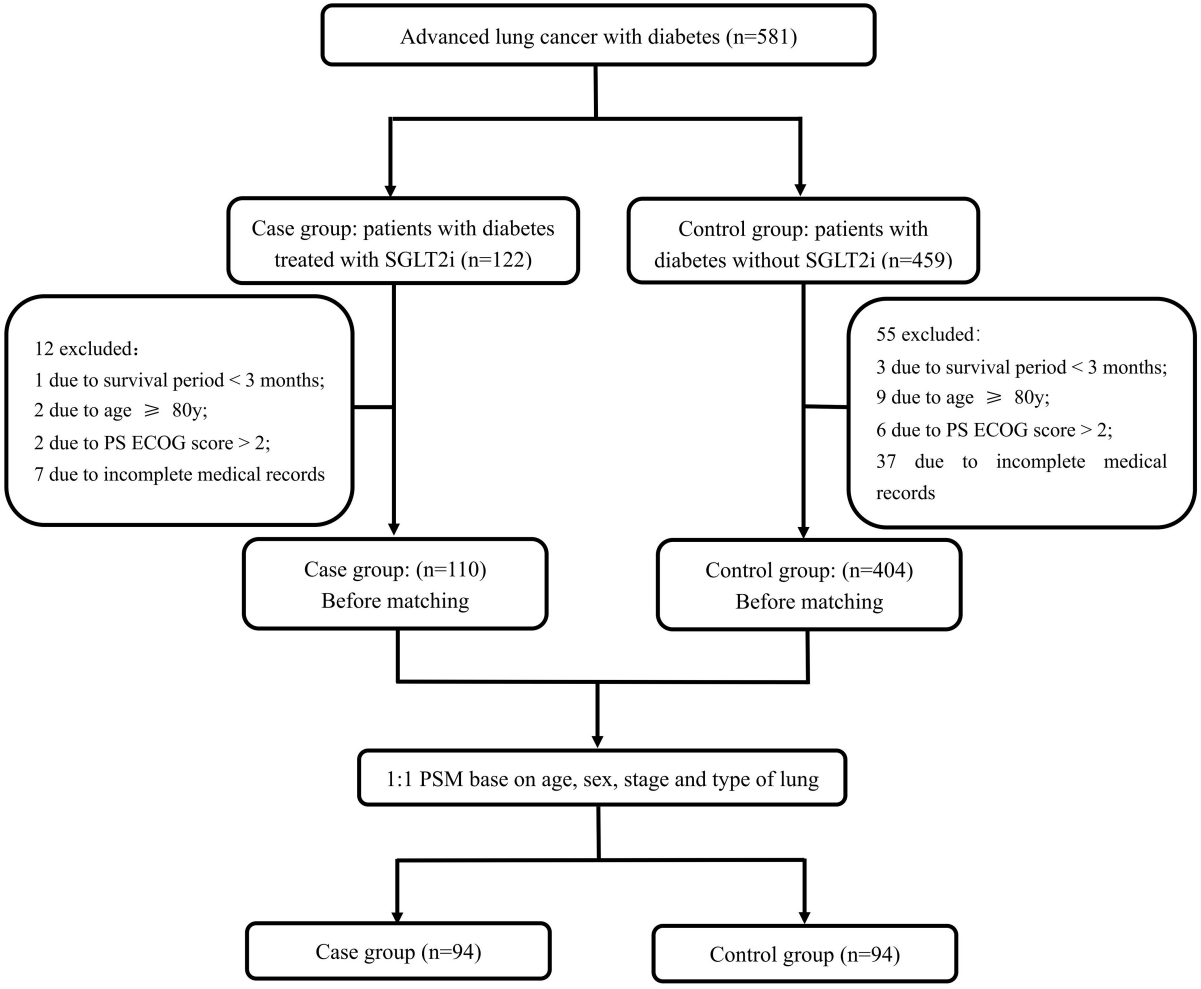
Flow diagram of patient selection process.

Among 581 advanced lung cancer with diabetes under chemotherapy, immunotherapy, targeted therapy, and/or radiotherapy, 94 patients under concomitant SGLT2i were included in the SGLT2i group and their matched 94 patients without SGLT2i treatment were included in the control group. The detailed selection process is illustrated in **Figure 1**. Among the 94 patients receiving SGLT2i therapy, the majority received a dosage of 10 mg **o**nce daily, and the median duration of treatment was 13 months (**Supplementary Table S1**).

Baseline clinical characteristics are summarized in **Table 1**. The groups were well matched at baseline. There were no significant differences in age, gender, cancer types, stages and therapy between the two groups. In cardiovascular risk factors, coronary artery disease was more prevalent in SGLT2i group than non- SGLT2i group (35.1% [33/94] vs. 17.0% [16/94]; P = 0.005). A similar trend was observed for insulin use (60.6% [57/94] vs. 44.7% [42/94]; P = 0.028). Hemoglobin A1C levels was higher in the SGLT2i group (7.5 ± 1.2% vs. 7.1 ± 1.3%; P = 0.040). No other statistically significant differences were observed in the remaining baseline variables (P>0.05 for all comparisons).

**Table 1:** Baseline characteristics of the cohort population.

Variables	Entire cohort (n=188)	SGLT2i (n=94)	non-SGLT2i (n=94)	P value
Demographics				
Age, years	65 ± 7	65 ± 8	66 ± 7	0.640
male	156 (82.9)	77 (81.9)	79 (84.0)	0.698
Cancer Types				0.487
NSCLC	167 (88.8)	82 (87.2)	85 (90.4)	
SCLC	21 (11.2)	12 (12.8)	9 (9.6)	
Cancer Stages				0.866
3	47 (25.0)	24 (25.5)	23 (24.5)	
4	141 (75.0)	70 (74.5)	71 (75.5)	
Anticancer therapy				
Chemotherapy	162 (86.2)	81 (86.2)	81 (86.2)	1.000
Radiotherapy	56 (29.8)	33 (35.1)	23 (24.5)	0.111
Immunotherapy therapy	114 (60.6)	63 (67.0)	51 (54.3)	0.073
Target therapy	71 (37.8)	33 (35.1)	38 (40.4)	0.452
Cardiovascular risk factors				
Hypertension	105 (55.9)	51 (54.3)	54 (57.4)	0.659
Coronary heart disease	49 (26.1)	33 (35.1)	16 (17.0)	0.005
Chronic kidney disease	9 (4.8)	4 (4.3)	5 (5.3)	0.733
Smoking	116 (61.7)	63 (67.0)	53 (56.4)	0.134
BMI	23.7 ± 2.9	23.9 ± 3.1	23.5 ± 2.6	0.356
Medications				
Beta blockers	58 (30.9)	34 (36.2)	24 (25.5)	0.114
ACEI/ARB/ARNI	65 (34.6)	38 (40.4)	27 (28.7)	0.092
Metformin	101 (53.7)	48 (51.1)	53 (56.4)	0.465
Insulin	99 (52.7)	57 (60.6)	42 (44.7)	0.028
Baseline laboratory parameters				
Hemoglobin A1C (%)	7.3 ± 1.2	7.5 ± 1.2	7.1 ± 1.3	0.040
Creatinine (ummol/L)	74 ± 20	74 ± 20	74 ± 20	0.678
CTnT (ng/mL)	0.014 ± 0.019	0.012 ± 0.013	0.016 ± 0.024	0.066

Data are presented as mean ± standard deviation or n (%). SGLT2i, Sodium-glucose cotransporter-2 inhibitors; NSCLC, non-small cell lung cancer; SCLC, small cell lung cancer; BMI, body-mass index; ACEI, angiotensin-converting enzyme inhibitor; ARB, angiotensin II receptor blocker; ARNI = angiotensin receptor-neprilysin inhibitors; CTnT = cardiac troponin T.

### 3.2 Anti-tumor treatment regimens

The specific cancer therapy protocol was chosen by oncologists according to current guidelines (11, 12). There was no statistically significant difference in the proportion of chemotherapy and targeted therapy between SGLT2i group and non-SGLT2i group. SGLT2i group had a higher proportion of patients receiving immunotherapy and radiotherapy, but without statistical significance.

### 3.3 Primary endpoint

**Figure 2:**
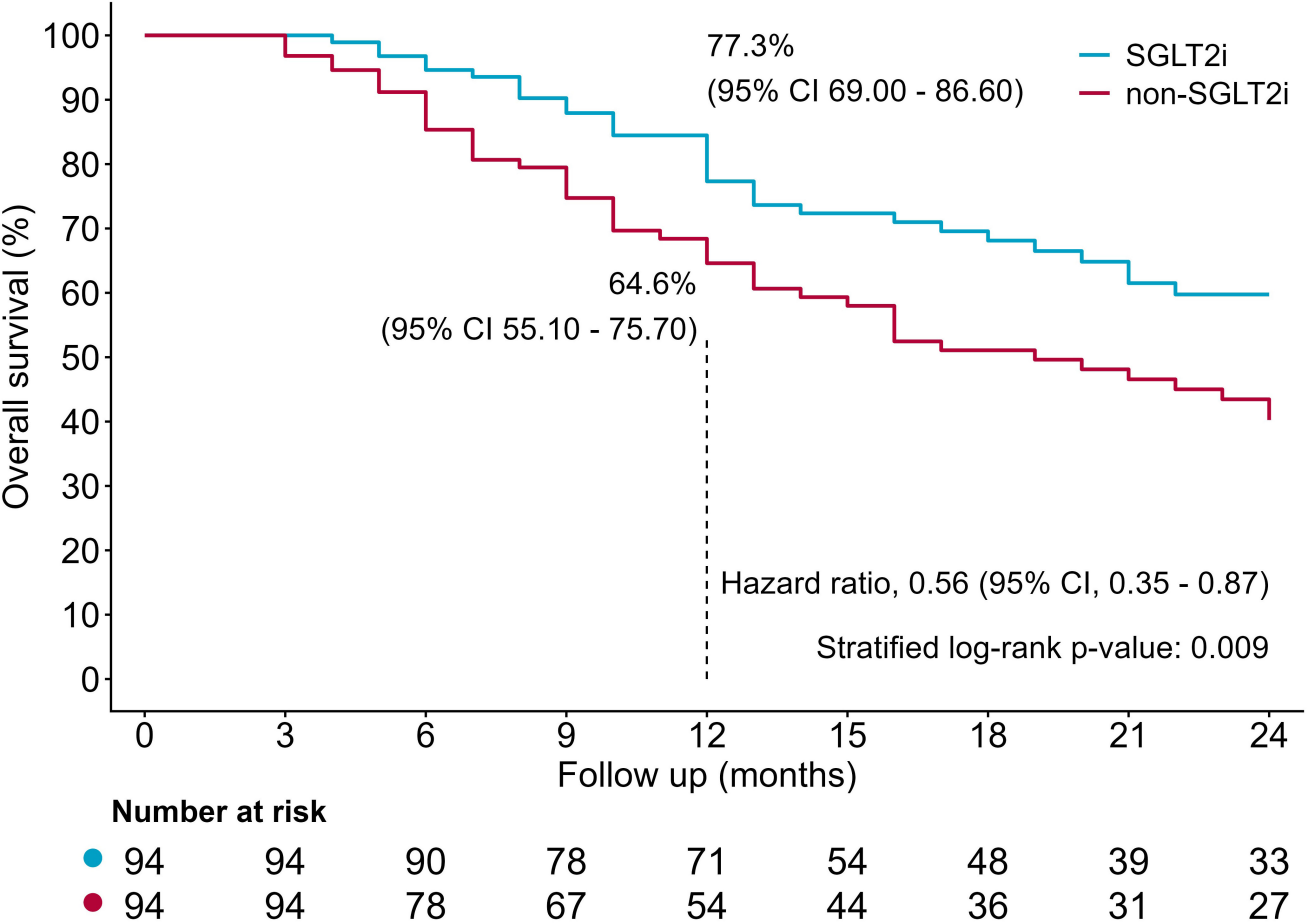
Kaplan-Meier curves between SGLT2i and non-SGLT2i.

**Figure 3:**
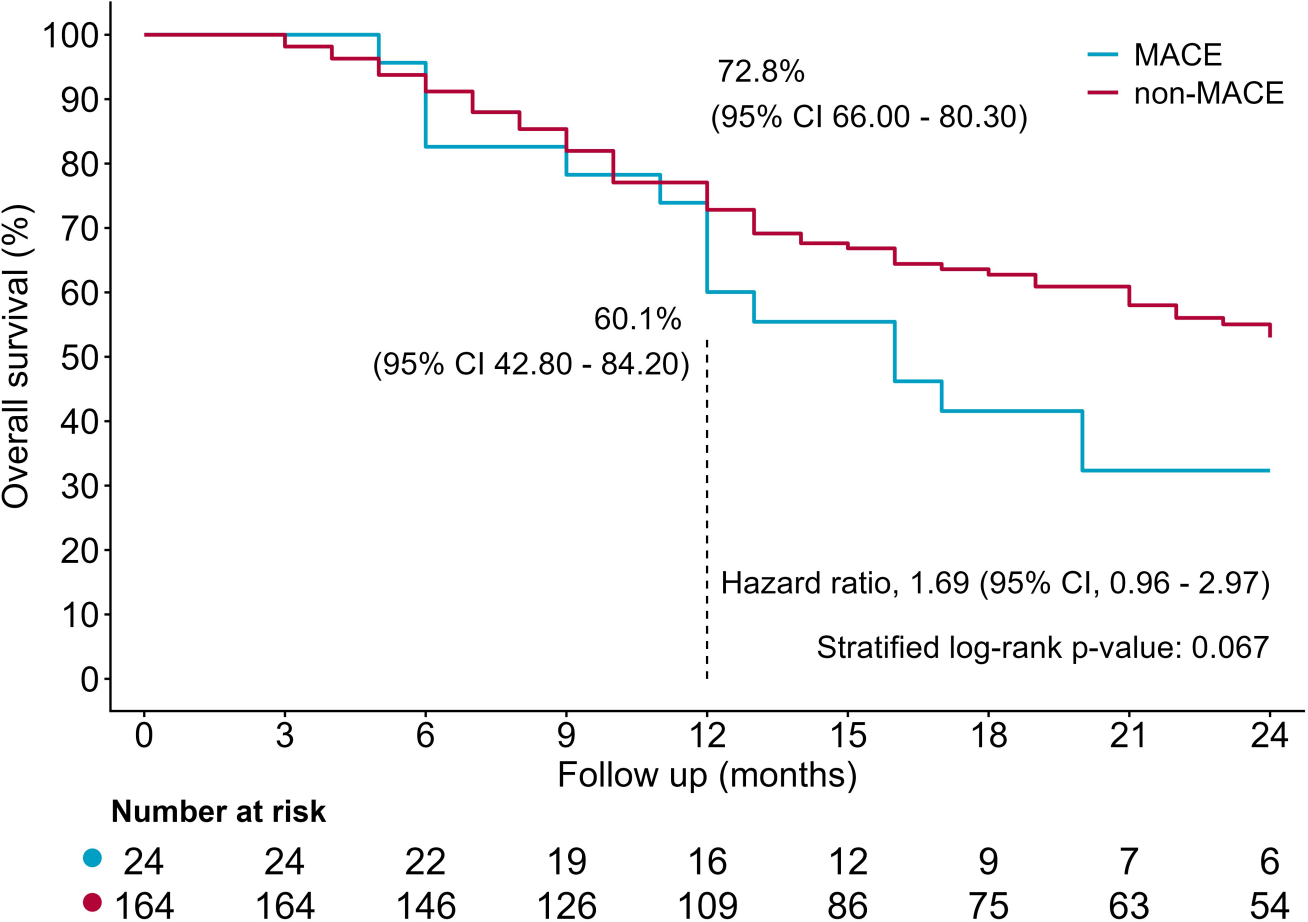
Kaplan-Meier curves between MACE and non-MACE.

Over a median follow-up of 16 months, 79 (42.0%) patients died, including 32 patients in SGLT2i group (32/94, 34.0%) and 47 in non-SGLT2i group (47/94, 50.0%). The all-cause mortality rate is significantly lower in SGLT2i group than in non-SGLT2i group (34.0% vs. 50.0%, P = 0.027, **Table 2**). Compared with non-SGLT2i group, SGLT2i group had a significantly improved outcome (HR = 0.56; 95% CI 0.35– 0.87, P = 0.009, **Figure 2**), with higher estimated survival rates at 12 months [77.3% (95% CI 69.00–86.60)vs. 64.6% (95% CI 55.10–75.70)] and 24 months [59.7% (95% CI 49.60–72.00) vs. 40.2% (95% CI 30.40–53.20)], as yielded by Kaplan-Meier analysis. During follow-up, patients with MACE had a lower survival rate at 12 months [60.1% (95% CI 42.80–84.20) vs. 72.8% (95% CI 66.00–80.30)] and 24 months [32.3% (95% CI 17.60–59.30) vs. 53.0% (95% CI 44.90–62.50)], compared with patients without MACE. However, the mortality risk was similar. (HR 1.69; 95% CI 0.96–2.97, P = 0.067, **Figure 3**). Univariate cox regression included age, sex, cancer types, stages, smoking, BMI, anticancer therapy, cardiovascular risk factors, medications, baseline laboratory parameters and SGLT2i. Variables with a P value < 0.05 including SGLT2i, ACEI/ARB/ARNI, smoking, and male were adjusted in the multivariable cox regression analysis (**Table 3**). After adjusting for confounding factors including HbA1c, history of coronary artery disease, and insulin use, multivariable cox regression analysis yielded that SGLT2i was an independent significant predictor for lower all-cause mortality (HR = 0.618; 95% CI 0.390–0.979, P = 0.041). After adjusting for confounding factors including HbA1c and history of coronary, multivariable cox regression analysis yielded that SGLT2i was an independent significant predictor for lower all-cause mortality (HR = 0.576; 95% CI 0.366–0.907, P = 0.017).

**Table 2:** Primary and secondary endpoints.

Endpoints	Entire cohort (n=188)	SGLT2i (n=94)	non-SGLT2i (n=94)	P value
All-cause Mortality	79 (42.0)	32 (34.0)	47 (50.0)	0.027
Composite cardiovascular outcomes	24 (12.8)	11 (11.7)	13 (13.8)	0.662
Atrial fibrillation/flutter	12 (6.4)	4 (4.2)	8 (8.5)	
Acute Coronary Syndrome	3 (1.6)	1 (1.1)	2 (2.1)	
Myocarditis	4 (2.1)	2 (2.1)	2 (2.1)	
Heart failure	8 (4.3)	4 (4.3)	4 (4.3)	
Third-degree AV block	1 (0.5)	1 (1.1)	0 (0)	

Values are n (%). Composite was defined by new-onset atrial fibrillation/flutter, acute coronary syndrome, myocarditis, heart failure and third-degree AV block. SGLT2i, Sodium-glucose cotransporter-2 inhibitors; AV block, atrioventricular block.

**Table 3:** Cox proportional hazards regression for all-cause mortality.

Variables	Univariate cox regression		Multivariable cox regression	
	P value	HR (95%CI)	P value	HR (95%CI)
SGLT2i	0.011	0.557 (0.356-0.874)	0.010	0.550 (0.347-0.869)
ACEI/ARB/ARNI	0.030	0.579 (0.353-0.949)	0.054	0.613 (0.373-1.009)
Male	0.037	2.178 (1.048-4.526)	0.204	1.732 (0.743-4.038)
Smoking	0.036	1.673 (1.035-2.705)	0.174	1.476 (0.841-2.589)

SGLT2i, sodium-glucose cotransporter-2 inhibitors.

### 3.4 Secondary endpoints

Overall, a total of 24 cardiovascular adverse events occurred, including 11 events in the SGLT2i group and 13 events in the control group, of which new-onset atrial fibrillation or atrial flutter occurred most frequently (n=12). In SGLT2i group, cardiovascular adverse events included atrial fibrillation/flutter in 4 patients, HF in 4 patients, myocarditis in 2 patients, ACS in 1 patient, and third-degree AV block in 1 patient. While in control group, there were atrial fibrillation/flutter in 8 patients, HF in 4 patients, myocarditis in 2 patients, ACS in 2 patients. No events of sustained ventricular tachycardia or ventricular fibrillation were recorded in either group. (**Table 2**). The MACE incidence rate was insignificant between the two groups (11.7% vs 13.8%; P = 0.662).

### 3.5 SGLT2i-related adverse events beyond cardiovascular adverse

Overall, the incidence of diabetes treatment-related adverse events was similar between SGLT2i group and non-SGLT2i group. The most frequent adverse event was acute kidney injury, which occurred in 6 patients in SGLT2i group and 12 patients in non-SGLT2i group (6.4% [6/94] vs. 12.8% [12/94]; P = 0.137). Urinary tract infection is the second most common adverse, which was observed in 3 patients in SGLT2i group and 2 patients in non-SGLT2i group. (3.2% [3/94] vs. 2.1% [2/94]; P = 0.650). Other adverse events included hypoglycemia in 2 patients in non-SGLT2i group and diabetic ketoacidosis in 1 patient in SGLT2i group.

## 4 Discussion

In this retrospective study, we assessed the impact of SGLT2i on the overall survival and cardiac outcome in patients with advanced lung cancer and diabetes. Our study reported three novel findings: 1) SGLT2i treatment was associate with a significantly lowered all-cause mortality in advanced lung cancer patients with diabetes; 2) The survival rate was lower in MACE group than non-MACE group, however the differences were not statistically significant; 3) The incidence of new-onset MACE tended to be lower in patients treated with SGLT2i, yet the results yielded no significant difference.

### 4.1 Cardioprotective effects of SGLT2i

Similar to the multiple roles of selenoproteins in diabetes (13), SGLT2i is also closely associated with both diabetes and heart disease. Although it remains undetermined about the detailed mechanism of the improved outcome associated with SGLT2i, it seems plausible that the cardioprotective effect of SGLT2i played a critical role in alleviating the cardiotoxicity of anti-cancer treatments. SGLT2i target the SGLT2 protein, which is located on the luminal border of the epithelial cells of the proximal convoluted tubules in the kidney. This cotransporter ordinarily reabsorbs 90–97% of the glucose filtered at the glomerulus (14). SGLT2i effectively treat type 2 diabetes by improving glucose levels, which are associated with reductions in body mass and blood pressure. Beyond diabetes, they also protect against heart and kidney complications, decreasing heart failure hospitalization and effect seen even in non-diabetic patients with reduced ejection fraction heart failure (15). In previous studies, SGLT2i could potentially mitigate the adverse effects, especially cardiotoxicity of cancer treatments (16, 17). The incidence of MACE was insignificant in the present study, which is consistent with previous studies (10). However, a trend toward fewer MACE was observed in SGLT2i group. The total number of cardiovascular events was relatively low, and our study may have been underpowered to detect a statistically significant difference in MACE rates, representing a potential type II error. Definitive evaluation of the cardioprotective effects of SGLT2i in this specific population requires future investigation with larger sample sizes and longer follow-up durations to adequately capture cardiovascular endpoints.

### 4.2 Potential anti-tumor effects: direct effects of SGLT2i

Other than cardioprotective effect, SGLT2i may also exert potential anti-tumor effect via multiple mechanisms, including metabolic modulation, disruption of oncogenic signaling pathways, and synergism with conventional therapies. 1) Reduction of glucose uptake in tumor cells. The Warburg effect, marked by elevated glucose consumption and lactate fermentation, is a prevalent characteristic of lung cancer (18). SGLT2 is one of the transporters for active glucose uptake into cancer cell, which has been detected in lung cancer (6, 7). SGLT2i reduces glucose uptake in cancer cells, thereby impairing their metabolic adaptability and proliferation. 2) Interference with tumor cell signaling pathways. Beyond metabolic effects, SGLT2i may disrupt critical oncogenic signaling cascades. Dapagliflozin has also been demonstrated to suppress tumor growth and clonogenic survival by targeting the AMPK/mTOR pathway in breast cancer (19). The AMPK/mTOR Pathway as a critical driver of lung cancer growth, potential modulation by dapagliflozin to suppress tumor progression and improve survival. It is noteworthy that these pathways also serve as core regulatory hubs in the development of diabetic complications, such as diabetic cardiomyopathy. Previous study indicates that dysregulation of signaling pathways including TGF-β/Smad, NF-κB, PI3K/AKT, Nrf2, and AMPK is a key mechanism underlying myocardial injury, and that multi-targeted interventions on these pathways can offer therapeutic benefits (20). 3) Synergistic effects with cancer therapy. The combination of SGLT2i with conventional anticancer therapies presents a promising therapeutic strategy. Current clinical evidence suggested that the addition of SGLT2i can enhance the efficacy of standard chemotherapy, radiotherapy targeted or immunotherapy in cancer patients (10, 21–23).

### 4.3 Potential anti-tumor effects: indirect effects of SGLT2i

Beyond the direct antitumor effects of SGLT2i, indirect mechanisms may also confer potential survival benefits. 1) Ameliorating metabolic derangements. The relationship between type 2 diabetes and cancer is shaped by several metabolic abnormalities, including hyperinsulinemia, elevated insulin-like growth factor I (IGF-I), hyperglycemia, and inflammatory cytokines (24). In addition, malignancies can disrupt glucose metabolism by inducing peripheral insulin resistance and sequestering glucose from skeletal muscle and adipose tissue to fuel tumor proliferation (25). Therefore the use of SGLT2i may offer significant benefits in improving survival outcomes by potentially ameliorate metabolic derangements. 2) Weight and blood pressure management. SGLT2i effectively manage body weight and blood pressure (26), with obesity and hypertension having been demonstrated in previous studies to contribute to disease progression in cancer patients (24). 3) Cardiorenal protective effects. SGLT2i exhibit potential cardiorenal protective effects while also mitigating cancer-therapy-induced cardiotoxicity (27).

### 4.4 SGLT2i without additional risks

In patients with advanced lung cancer receiving medical therapy, acute kidney injury was not an uncommon complication. Although there was no significant difference in baseline creatinine levels between the two groups and the difference in the incidence of acute kidney injury did not reach statistical significance, we observed a numerically lower incidence of acute kidney injury in the SGLT2i group compared with the control group. This trend may be related to the known reno-protective mechanisms of SGLT2i. Through multiple pathways—such as reducing intraglomerular pressure, improving tubular hypoxia, attenuating inflammation and fibrosis, direct cellular protection and systemic benefits with indirect renal effects (28). In this study, patients with advanced lung cancer are often at high risk of acute kidney injury due to factors such as the tumor itself, dehydration, or nephrotoxic agents. The potential reno-protective effects of SGLT2i may have partially mitigated the impact of these factors on renal function. However, it must be emphasized that, given the limited sample size and retrospective design of this study, this observation should be interpreted as preliminary and may be influenced by unmeasured confounding factors. Future larger-scale prospective studies are warranted to specifically evaluate the renal safety and protective effects of SGLT2i in cancer patients, particularly those receiving nephrotoxic anticancer therapies. Interestingly, the SGLT2i group showed a numerically lower incidence compared with the control group, although the difference did not reach statistical significance. This observation may reflect potential renal protective effects of SGLT2i, consistent with their known mechanisms of action (29). SGLT2i promote urinary glucose excretion, a mechanism that has been associated with an increased risk of urinary tract infections (30). In prior studies, urinary tract infection was the most frequently reported adverse effect of SGLT2i, with an incidence of approximately 3.6-5.7% (29). In our study, the incidence of urinary tract infections was 3.2% in patients with SGLT2i was similar to previous reports (29). In addition, compared with the control group, SGLT2i did not significantly increase the risk of urinary tract infections. The incidence of hypoglycemia and ketoacidosis was low in both the SGLT2i and control groups without significant difference. Our findings suggest that the use of SGLT2i did not increase the risk of these adverse events.

### 4.5 Diabetic survival benefits and non-diabetic knowledge gaps

The demonstrated efficacy of SGLT2i in improving survival rates among patients with advanced lung cancer and diabetes, without increasing additional risks, could support their integration into treatment protocols for this vulnerable population. Current standard cancer therapies, including chemotherapy and immunotherapy, may benefit from the adjunctive use of SGLT2i to optimize diabetic patient outcomes. However, whether SGLT2i confer survival benefits in non-diabetic patients with advanced lung cancer remains uncertain and warrants further investigation.

## 5 Study limitation

The present study has a few limitations. First, this is a retrospective observational study, and confounding factors may influence the results. Second, the number of observed MACE was relatively low, which limits the statistical power to detect significant differences in cardiovascular outcomes between groups. Moreover, the detailed mechanism currently remains unclear, which requires in-depth mechanism study in the future. Further prospective studies are warranted to verify the findings of the present study. Finally, our cohort was limited to diabetic individuals. Although SGLT2i show efficacy in diabetic cancer populations, their potential role in non-diabetic cancer patients requires dedicated study.

## 6 Conclusion

The use of SGLT2i was associated with a lower rate of all-cause mortality in advanced lung cancer patients with diabetes. SGLT2i had insignificant impact on the incidence of MACE and the post-MACE survival. Further studies are needed to verify the cardioprotective effect of SGLT2i in this subgroup of patients.

## Data Availability

The raw data supporting the conclusions of this article will be made available by the authors, without undue reservation.
